# Urbanization: a problem for the rich and the poor?

**DOI:** 10.1186/s40985-019-0116-0

**Published:** 2020-01-02

**Authors:** Md Abdul Kuddus, Elizabeth Tynan, Emma McBryde

**Affiliations:** 1 Australian Institute of Tropical Health and Medicine, James Cook University, Townsville, Australia; 2 College of Medicine and Dentistry, James Cook University, Townsville, Australia; 3 Graduate Research School, James Cook University, Townsville, Australia; 4 Department of Mathematics, University of Rajshahi, -6205, Rajshahi, Bangladesh

**Keywords:** Urbanization, Diseases, Public health, Nutrition

## Abstract

Urbanization has long been associated with human development and progress, but recent studies have shown that urban settings can also lead to significant inequalities and health problems. This paper is concerned with the adverse impact of urbanization on both developed and developing nations and both wealthy and poor populations within those nations, addressing issues associated with public health problems in urban areas. The discussion in this paper will be of interest to policy makers. The paper advocates policies that improve the socio-economic conditions of the urban poor and promote their better health. Further, this discussion encourages wealthy people and nations to become better informed about the challenges that may arise when urbanization occurs in their regions without the required social supports and infrastructure.

## Main text

Urbanization refers to the mass movement of populations from rural to urban settings and the consequent physical changes to urban settings. In 2019, the United Nations estimated that more than half the world’s population (4.2 billion people) now live in urban area and by 2041, this figure will increase to 6 billion people [1].

Cities are known to play multifaceted functions in all societies. They are the heart of technological development and economic growth of many nations, while at the same time serving as a breeding ground for poverty, inequality, environmental hazards, and communicable diseases [2]. When large numbers of people congregate in cities, many problems result, particularly for the poor. For example, many rural migrants who settle in an urban slum area bring their families and their domesticated animals—both pets and livestock—with them. This influx of humans and animals leads to vulnerability of all migrants to circulating communicable diseases and the potential to establish an urban transmission cycle. Further, most urban poor live in slums that are unregulated, have congested conditions, are overcrowded, are positioned near open sewers, and restricted to geographically dangerous areas such as hillsides, riverbanks, and water basins subject to landslides, flooding, or industrial hazards. All of these factors lead to the spread of communicable and non-communicable diseases, pollution, poor nutrition, road traffic, and so on [3–5]. The problems faced by the poor spill over to other city dwellers. As the trend to urbanization continues, this spillover effect increases and takes on a global dimension as more and more of the world’s populations are affected [3].

Some of the major health problems resulting from urbanization include poor nutrition, pollution-related health conditions and communicable diseases, poor sanitation and housing conditions, and related health conditions. These have direct impacts on individual quality of life, while straining public health systems and resources [6].

Urbanization has a major negative impact on the nutritional health of poor populations. Because they have limited financial resources and the cost of food is higher in cities, the urban poor lack nutritious diets and this leads to illness, which contributes to loss of appetite and poor absorption of nutrients among those affected. Furthermore, environmental contamination also contributes to undernutrition; street food is often prepared in unhygienic conditions, leading to outbreaks of food-borne illnesses (e.g., botulism, salmonellosis, and shigellosis) [6]. Urban dwellers also suffer from overnutrition and obesity, a growing global public health problem. Obesity and other lifestyle conditions contribute to chronic diseases (such as cancers, diabetes, and heart diseases). Although obesity is most common among the wealthy, international agencies have noted the emergence of increased weight among the middle class and poor in recent years [7].

Populations in poor nations that suffer from protein-energy malnutrition [8] have increased susceptibility to infection [9] through the impact of micronutrient deficiency on immune system development and function [10]. Around 168 million children under 5 are estimated to be malnourished and 76% of these children live in Asia [11]. At the same time, the World Health Organization is concerned that there is an emerging pandemic of obesity in poor countries that leads to non-communicable diseases such as diabetes, cardiovascular disease, cancer, hypertension, and stroke [12].

Obesity is caused by increased caloric intake and decreased physical activity [13], something historically associated with wealth. However, people in urbanized areas of developing countries are also now vulnerable to obesity due to lack of physical space, continually sitting in workplaces, and excessive energy intake and low energy expenditure. In these areas, infrastructure is often lacking, including sufficient space for recreational activities. Further, in developing countries, as in developed countries, large employers frequently place head offices in urban capitals and work is increasingly sedentary in nature [14]. Another culprit associated with the risk of developing obesity is the change in food intake that has led to the so-called nutrition transition (increased the consumption of animal-source foods, sugar, fats and oils, refined grains, and processed foods) in urban areas. For instance, in China, dietary patterns have changed concomitantly with urbanization in the past 30 years, leading to increased obesity [15]. In 2003, the World Health Organization estimated that more than 300 million adults were affected, the majority in developed and highly urbanized countries [16]. Since then, the prevalence of obesity has increased. For example, in Australia, around 28% of adults were obese in 2014–2015 [17].

Pollution is another major contributor to poor health in urban environments. For instance, the World Health Organization estimated that 6.5 million people died (11.6% of all global deaths) as a consequence of indoor and outdoor air pollution and nearly 90% of air-pollution-related deaths occurred in low- and middle-income countries [18]. Poor nutrition and pollution both contribute to a third major challenge for urban populations: communicable diseases. The poor live in congested conditions, near open sewers and stagnant water, and are therefore constantly exposed to unhealthy waste [6]. Inadequate sanitation can lead to the transmission of helminths and other intestinal parasites. Pollution (e.g., from CO_2_ emission) from congested urban areas contributes to localized and global climate change and direct health problems, such as respiratory illnesses, cardiovascular diseases, and cancer for both the rich and the poor.

In addition to human-to-human transmission, animals and insects serve as efficient vectors for diseases within urban settings and do not discriminate between the rich and poor. The prevalence and impact of communicable diseases in urban settings, such as tuberculosis (TB), malaria, cholera, dengue, and others, is well established and of global concern.

National and international researchers and policy makers have explored various strategies to address such problems, yet the problems remain. For example, research on solutions for megacities has been ongoing since the early 1990s [19, 20]. These studies have concluded that pollution, unreliable electricity, and non-functioning infrastructure are priority initiatives; nevertheless, air pollution, quality of water in cities, congestion, disaster management issues, and infrastructure are not being systematically addressed [19, 20].

The impact of inner city transportation on health, such as road traffic, is emerging as a serious problem. Statistics show that a minimum of 10 people die every day on the railways in the city of Mumbai, India [21]. Vietnam is another example of a country that has seen a remarkable increase in road traffic accidents [22]. Improvements to the country’s infrastructure have not been able to meet the increasing growth of vehicular and human traffic on the street. Vietnam reportedly has a population of 95 million and more than 18 million motorbikes on its roads. A deliberate policy is needed to reduce accidents [21].

Although urbanization has become an irreversible phenomenon, some have argued that to resolve the problems of the city, we must tackle the root causes of the problem, such as improving the socio-economic situation of the urban poor.

Until the conditions in rural areas improve, populations will continue to migrate to urban settings. Given the challenges that rural development poses, the root causes are unlikely to be addressed in the near future. Therefore, governments and development agencies should concentrate on adapting to the challenges of urbanization, while seeking to reduce unplanned urbanization.

Some examples of policies and practices that should be considered include (i) policies that consider whole-of-life journeys, incorporating accessible employment, community participation, mobility/migration and social transition, to break generational poverty cycles; (ii) policies addressing urban environmental issues, such as planned urban space and taxes on the use of vehicles to reduce use or to encourage vehicles that use less fuel as well as encourage bicycle use, walking, and other forms of human transportation; (iii) greater cooperative planning between rural and urban regions to improve food security (e.g., subsidies for farmers providing locally produced, unprocessed and low cost food to urban centers); (iv) social protection and universal health coverage to reduce wealth disparity among urban dwellers; including introduction of programs and services for health, for example by establishing primary healthcare clinics accessible and affordable for all including those living in urban slums [23].

## Data Availability

Not applicable
